# Linking Brain Structure, Activity, and Cognitive Function through Computation

**DOI:** 10.1523/ENEURO.0316-21.2022

**Published:** 2022-03-11

**Authors:** Katrin Amunts, Javier DeFelipe, Cyriel Pennartz, Alain Destexhe, Michele Migliore, Philippe Ryvlin, Steve Furber, Alois Knoll, Lise Bitsch, Jan G. Bjaalie, Yannis Ioannidis, Thomas Lippert, Maria V. Sanchez-Vives, Rainer Goebel, Viktor Jirsa

**Affiliations:** 1 Institute of Neurosciences and Medicine (INM-1), Research Centre Jülich, Jülich 52425, Germany; 2 C. & O. Vogt Institute for Brain Research, University Hospital Düsseldorf, Heinrich-Heine University Düsseldorf, Düsseldorf 40225, Germany; 3 Laboratorio Cajal de Circuitos Corticales, Centro de Tecnología Biomédica, Universidad Politécnica de Madrid, Madrid 28223, Spain; 4 Instituto Cajal, Consejo Superior de Investigaciones Científicas (CSIC), Madrid 28002, Spain; 5 Cognitive and Systems Neuroscience Group, Swammerdam Institute for Life Sciences, University of Amsterdam, Amsterdam, 1098 XH, The Netherlands; 6 Centre National de la Recherche Scientifique, Institute of Neuroscience (NeuroPSI), Paris-Saclay University, Gif sur Yvette 91400, France; 7 Institute of Biophysics, National Research Council, Palermo 90146, Italy; 8 Department of Clinical Neurosciences, Centre Hospitalier Universitaire Vaudois, Lausanne CH-1011, Switzerland; 9 Department of Computer Science, The University of Manchester, Manchester M13 9PL, United Kingdom; 10 Department of Informatics, Technical University of Munich, Garching 385748, Germany; 11 The Danish Board of Technology Foundation, Copenhagen, 2650 Hvidovre, Denmark; 12 Institute of Basic Medical Sciences, University of Oslo, Oslo, Norway; 13 ATHENA Research & Innovation Center, Athena 12125, Greece; 14 Department of Informatics & Telecom, Nat’l and Kapodistrian University of Athens, 157 84 Athens, Greece; 15 Institute for Advanced Simulation (IAS), Jülich Supercomputing Centre (JSC), Research Centre Jülich, Jülich 52425, Germany; 16 ICREA and Systems Neuroscience, Institute of Biomedical Investigations August Pi i Sunyer, Barcelona 08036, Spain; 17 Department of Cognitive Neuroscience, Department of Cognitive Neuroscience, Faculty of Psychology and Neuroscience, Maastricht University, Maastricht 6229 EV, The Netherlands; 18 Aix Marseille Université, Institut National de la Santé et de la Recherche Médicale, Institut de Neurosciences des Systèmes (INS) UMR1106, Marseille 13005, France

**Keywords:** artificial neuronal networks, brain complexity, connectivity, human brain mapping, multiscale brain organization, neuro-inspired technology

## Abstract

Understanding the human brain is a “Grand Challenge” for 21st century research. Computational approaches enable large and complex datasets to be addressed efficiently, supported by artificial neural networks, modeling and simulation. Dynamic generative multiscale models, which enable the investigation of causation across scales and are guided by principles and theories of brain function, are instrumental for linking brain structure and function. An example of a resource enabling such an integrated approach to neuroscientific discovery is the BigBrain, which spatially anchors tissue models and data across different scales and ensures that multiscale models are supported by the data, making the bridge to both basic neuroscience and medicine. Research at the intersection of neuroscience, computing and robotics has the potential to advance neuro-inspired technologies by taking advantage of a growing body of insights into perception, plasticity and learning. To render data, tools and methods, theories, basic principles and concepts interoperable, the Human Brain Project (HBP) has launched EBRAINS, a digital neuroscience research infrastructure, which brings together a transdisciplinary community of researchers united by the quest to understand the brain, with fascinating insights and perspectives for societal benefits.

## Visual Abstract

**Figure:**
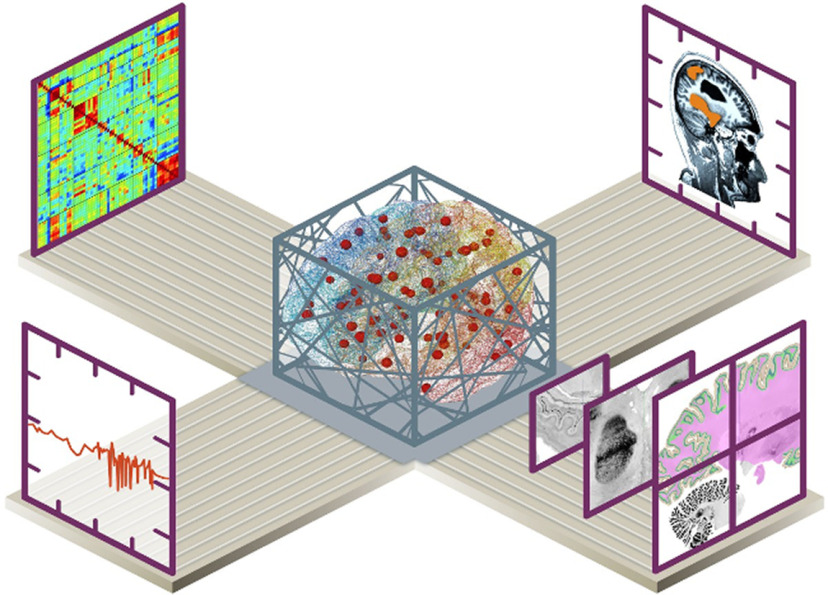

## Significance Statement

Theoretical and methodological integration leads to consolidation and deeper intuitive understanding, which is required for coherent and systematic scientific progress. In 2013, the European Union launched the Human Brain Project (HBP) with the mission to integrate spatial and temporal scales of brain sciences within a common framework, ultimately leading to the digital research infrastructure EBRAINS. It has become evident that doing science in EBRAINS will require a culture change in the neuroscientific community, a transformation that has already been observed in large-scale projects of other scientific disciplines such as elementary particle physics. Novel HBP-style neuroscience is characterized by transparent domain boundaries and deep integration of highly heterogeneous data, models, and information technologies. In this article HBP scientists present their scientific approach and illustrate the exciting potential of the EBRAINS ecosystem for neuroscience research.

## Introduction

Advances in science have been driven by the human search for knowledge and understanding of nature, from the world around us to principles governing the whole universe. But there is a universe inside each one of us that manifests and defines our consciousness, cognition, behavior, emotions, health and illness, a universe that remains relatively unexplored yet contains the secrets of our human nature. It gives rise to behavior that we are all familiar with, allowing us to communicate, but also to manipulate information, be creative and spontaneous, make informed decisions, reason about moral and ethical questions and much more. Human curiosity has driven researchers forward to search for knowledge and understanding of this universe, which is per se a legitimate human endeavor. This search, however, is most challenging because of the complexity of the brain. Similar to research into other complex systems, brain research benefits from computational analysis tools as well as from new forms of collaboration, including large national and international consortia. Compared with other research disciplines such as nuclear physics or astronomy, such large-scale collaboration is not so common in the fields of neuroscience and medicine. It is, however, not by chance that large national and international projects devoted to brain investigation have surfaced around the world in the last decade (Adams et al., 2020; Quaglio et al., 2021).

The present article will:

- Provide a brief overview of the present status of key aspects of brain research and related challenges towards a deeper understanding of brain complexity
- Motivate research focused on the multilevel organization of the brain, both in space and time, and to better understand the rules by which observations at a lower scale influence those at the higher one, and vice versa
- Highlight the role of theory, brain modelling and simulation to explore the multiscale organization of the brain
- Argue for the need to develop new tools for data analytics, brain-inspired learning, neurorobotics and atlasing of the brain under a common roof, i.e., a joint research infrastructure
- Elucidate how the European Human Brain Project (HBP) is contributing to brain research and why it is developing EBRAINS as a new research infrastructure, in a co-design approach between neuroscientists and developers, engineers and informaticists
- Indicate the perspectives for brain medicine arising therefrom
- Illustrate the potential for the development of brain-inspired computing, technology and high-performance computing
- Emphasize collaborative approaches
- Provide conclusions for future research

## Brain Complexity

The human brain is organized across different spatial scales, from molecules in the Ångström and nanometer range, to cells on micrometer scales, local neuronal circuits, to whole-brain networks at the centimeter scale, and functional systems underlying, for example, cognition and consciousness. Even though each level is unique in its organization of constituents and their activities, first principles nevertheless exist and account for functional or computational architectures that hold at multiple scales. Examples of this are the free energy principle and “synergetics” that explain self-organization and pattern formation at multiple scales (Haken, 1983; Kiebel and Friston, 2011; Huys et al., 2014; Friston et al., 2015, 2017). When modeling, the principles provide guidance realizing the computational processes and optimizing neuroanatomical and neurochemical structures, and the data provide the building blocks for the microcircuitry and networks across spatial and temporal dimensions. For instance, molecules may change their conformation within a few milliseconds, while other processes occur during the whole lifespan, over many decades.

Thus, functional architectures in the brain can be conceptualized at different scales of spatiotemporal organization, wherein molecular and cellular processes are subsumed under macroscopic functional entities like multiarea brain systems influencing behavior. Nerve cells are key components within this multilevel organization, and are themselves intricate autonomous structures, with a nucleus hosting genetic information, organelles involved in the production of proteins and metabolism, bilipid membranes in which receptors and other molecules are embedded, and trees of axons and dendrites with spines. The activities of most of these constituents, if not all, are organized in networks establishing a set of causal interactions, the interactome (Klein et al., 2021). Distinct anatomic networks display a hierarchical architecture with multiple nodes of convergence of afferents and divergence of efferents, providing the substrate for both serial and parallel processing. Furthermore, neuronal circuit activity with excitatory and inhibitory mechanisms of signal transduction is highly influenced by neuromodulators (e.g., serotonin, acetylcholine, and dopamine). These neuromodulators are secreted by groups of neurons located in the basal forebrain and brainstem, and reach large regions of the brain, where they may act either via release from non-synapsing varicosities and extracellular diffusion or via synaptic junctions on specific neuronal populations.

The functional significance of the various types of overall human brain connectivity has been explored thanks to the development of neuroimaging and neurophysiological techniques as well as mathematical models. In particular, investigating the complete network of anatomically interconnected brain regions, the connectome (Sporns et al., 2005), and its relationship with functional brain networks [using, for example, structural and functional MRI, magnetoencephalography, and electroencephalography (EEG)], has provided important advances in our knowledge of the general principles of structural and functional network organization of the human brain. In this regard, three types of connections are commonly recognized: (1) structural or anatomic connectivity; (2) functional connectivity, defined as statistical associations or dependencies between neurophysiological events recorded in distant brain regions; and (3) effective connectivity, defined as directed or causal relationships between brain regions (Bullmore and Sporns, 2009; Friston, 2011). Connectivity also evolves over time on multiple time scales (Hansen et al., 2015; Galadí et al., 2021) and establishes a functional connectivity dynamics predictive of aging (Battaglia et al., 2020; Escrichs et al., 2021), cognitive processes (Lombardo et al., 2020), and brain disease (Courtiol et al., 2020).

Neurons can be seen as central elements of a whole cascade of signal transduction, encompassing processes from the properties of ion channels up to the emergence of large-scale activity states (Goldman et al., 2019). For example, the apical dendrites of pyramidal neurons integrate information from a large dendritic network, and may serve as gates or switches, enabling or breaking global brain dynamics and regulating information flow, therefore potentially having a central role in the mechanism of consciousness (Aru et al., 2020). According to this view, during conscious processing, the bottom-up information stream would be integrated at the apical dendrite with a top-down stream, putting into focus the role of large networks and cognitive processes.

On the largest scales, information processing capacity is characterized by the network’s topochronic organization (Jirsa, 2008; Petkoski and Jirsa, 2019, 2022) as defined by the connectome’s strength and signal transmission delays, constraining the emergence of brain functions, for instance, in the emergence of consciousness. The global neuronal workspace theory of consciousness is a concrete manifestation thereof and emphasizes the role of frontoparietal networks (Dehaene and Changeux, 2011). This theory is compared with other information-theory-based (Tononi and Koch, 2015) and representational (Pennartz, 2015; Pennartz et al., 2019b) frameworks emphasizing the role of more posterior networks in, for instance, conscious vision, touch, and hearing. Large-scale corticothalamic networks and the complexity of their dynamics play a major role in the levels of consciousness and their quantification, critical both for basic brain mechanistic understanding (Llinás et al., 1998; Sanchez-Vives et al., 2017; Barbero-Castillo et al., 2021) and for clinical application, as in disorders of consciousness (Storm et al., 2017; Demertzi et al., 2019; Comanducci et al., 2020). The topic of bottom-up versus top-down perspectives in understanding multilevel brain organization has been intensively discussed in the past. It has been argued that a detailed bottom-up reconstruction and simulation of neuronal elements may reveal canonical microcircuits and reproduce results of *in vivo* experiments from which the laws of brain function will emerge (Markram et al., 2015). Along the same line of reasoning, it has been speculated that neuronal assemblies with their synaptic connections serve as innate, “Lego-like” building blocks of knowledge for perception and that the acquisition of memories involves the combination of these building blocks into complex constructs (Markram and Perin, 2011).

It is still a major challenge to explore how the different spatial scales are connected, for example, how precisely the binding of a neurotransmitter to its receptor modulates the activity of cell assemblies and large-scale networks involving long-distance fiber tracts and brain areas, from which, in the end, behavior emerges. Other questions are what the rules are that govern the underlying networks, and how it is possible that they are so effective and so efficient when they use so little energy. Likewise, much work remains to be done to elucidate how the brain interacts with the natural and cultural environment, e.g., how epigenetic mechanisms act on the brain, how genotype-phenotype relationships are linked with variations between brains and behavior, why aging or brain diseases affect some people more than others, and what determines the individual vulnerability to brain diseases.

Here, the top-down approach complements the strategy by using computational models as observation models that are fit to biological data (Friston, 2011; Huys et al., 2014; Pillai and Jirsa, 2017). These observational models effectively generate the data one would observe if the implicit generative model were correct. The explicit generative models establish a causal hypothesis, which uses the data to optimize the structure and parameters of some hypothetical network model, and evaluate the evidence for different models given the data. This dual approach guides the identification of causal mechanisms, going beyond the estimation of statistical correlations in traditional data mining approaches. Examples include the Perturbation Complexity Index (PCI) used to assess effective connectivity (Comolatti et al., 2019), variants of dynamic causal modeling used in The Virtual Brain (TVB; see below for examples of clinical applications) and uses of generative models in a “digital twin” approach (Hashemi et al., 2020; Vattikonda et al., 2021), which optimizes parameters to best explain personalized data as a prelude to characterizing within and between subject variability.

Many researchers converge on the notion that the two perspectives are not mutually exclusive and, even more, that bottom up-approaches need to be supplemented by conceptual approaches reducing structural complexity (Frégnac and Bathellier, 2015) and principled approaches making use of theories of brain function (Friston, 2011; Huys et al., 2014; Pillai and Jirsa, 2017). It has been argued to go beyond a simplistic top-down and bottom-up dichotomy, and to link the cognitive and brain perspectives (Ramsey and Ward, 2020). The unparalleled complexity of the brain may seem like a daunting challenge for any research project in the field, but it is a critical factor for the brain to organize itself and for the emergence of brain function and behavior. Cognition and behavior cannot be explained and predicted by the brain’s individual components alone. Instead, both so-called bottom-up and top-down approaches are necessary to understand brain organization, its role in signal transduction, cognitive processing and behavior. Information processing at axonal level is highly parallel, and at the same time characterized by both convergence and divergence (Rockland, 2020). It has been hypothesized that the laminar differentiation and the large number of neurons and areas, in combination with other factors, are key for cognitive abilities (Pennartz et al., 2019a, b; Changeux et al., 2021).

Finally, a multiscale comprehensive understanding of cognitive function and behavior at the end requires not only to link the cellular with the cognitive perspective, but also to include intermediate levels of information processing such as areas and cortical columns. An example are columnar clusters in the human motion complex reflecting specific contents of consciousness (Schneider et al., 2019). Such clusters are components of the brain’s organization into areas, layers, and other microstructural variations within areas (Amunts and Zilles, 2015; Amunts et al., 2020). Examples are giant Betz cells in the internal pyramidal cell layer of primary motor cortex, which give rise to long-range projections to the spinal cord, and the very broad and differentiated Layer IV in the primary visual cortex, receiving massive input from the retina via the lateral geniculate body.

Thus, laminar patterns reflect connectivity (Rockland and DeFelipe, 2018) and suggest a specific role of an area in a network, e.g., underlying cognitive functions and consciousness (Goulas et al., 2018). The concept of the “localization of function” is >100 years old. It was inspired by early physiological and lesion studies such as pioneered by Broca (Broca, 1861), Campbell (Campbell, 1905), the Vogts (Vogt and Vogt, 1926), and Foerster (Foerster, 1934), which observed clinical symptoms, behavioral or brain activity changes, that were specific for a certain brain region. These studies were complemented by studies targeting disconnection syndromes, e.g., by Karl Wernicke, who studied brains with language deficit after brain lesion (Wernicke, 1874; Lichtheim, 1885). This concept integrates the network perspective with the perspective of brain regions critically involved in language, and proposed the first comprehensive theory of language. Structure-function relationships at the level of brain areas play an important role in modern neuroimaging, and are incorporated in recent concepts of brain segregation and integration (Eickhoff et al., 2018).

**Figure 1.**
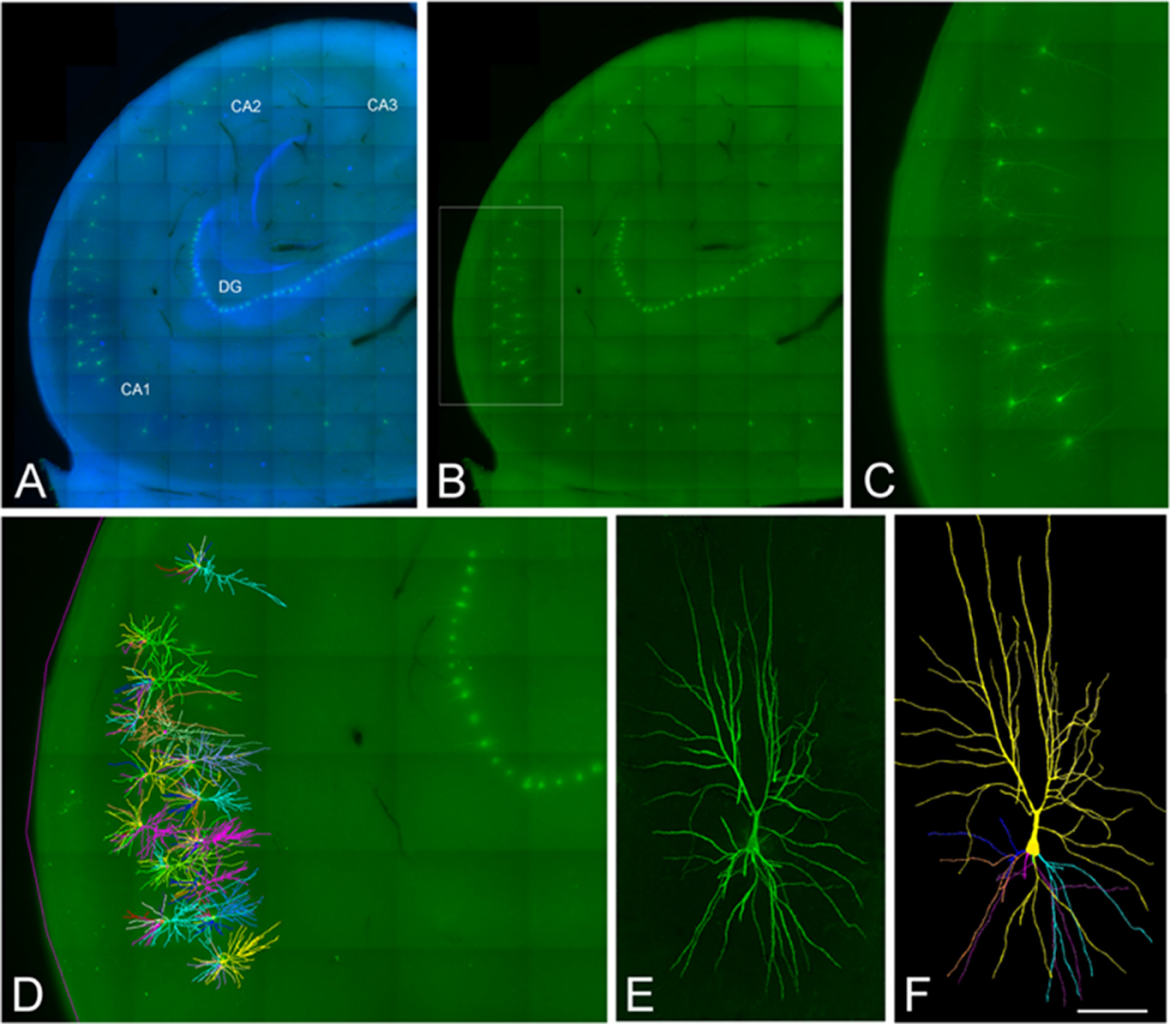
Confocal microscopy images of human neurons injected with Lucifer yellow in the hippocampus. ***A***, ***B***, Labeled pyramidal cells (green) and DAPI staining (blue) in different regions of the human hippocampus, including CA1, CA2, CA3, and the dentate gyrus region (DG). ***C***, Higher magnification image of the boxed region shown in ***B***. ***D***, 3D reconstructed cells superimposed on the confocal image shown in ***C***. ***E***, ***F***, High-magnification image z projection showing an injected CA1 pyramidal cell (***E***) and the 3D reconstruction of the same cell (***F***). Scale bar: 1100 μm (***A***, ***B***), 460 μm (***C***, ***D***), and 100 μm (***E***, ***F***). Image taken from Benavides-Piccione et al. (2020).

The comparison between species demonstrates that differences in brain organization are not simply a result of scaling as an effect of evolution, but are accompanied by changes in organization and complexity. A challenge results from the size of the human brain, and its increasing complexity. Major factors comprise, among others, the highly folded cerebral cortex, e.g., as compared with lisencephalic brains of rodents, the high degree of intersubject variability, and the large number of nerve cells, which is estimated to be 86 billion (Box 1; Fig. 1), as well as a greater molecular diversity of cell types (Hodge et al., 2019; Bakken et al., 2021; Berg et al., 2021).

Box 1: The human brain in numbers and examples to illustrate their magnitudes

Estimated number of nerve cells: ∼86 billion, approximately the same number of glial cells, ∼10,000 synapses per neuron; for comparison, a galaxy has ∼100 billion stars.

Type of signal transduction: electro-chemical with nerve conduction velocity between 1 and 100 m/s, while the speed of sound is ∼343 m/s.

Total length of connections: 2–3 million kilometers of fibers; for comparison, this is more than the diameter of the sun with 1.4 million kilometers

Mass: 1200–1500 g, i.e., ∼2% of the body weight

Energy consumption: 20–30 W, i.e., ∼20% of the total energy consumption of the body

The large size of the human brain with its complex organization is reflected at the level of data that describe it (Box 2). While a digitized mouse brain with 1-μm spatial resolution has a total volume of uncompressed data of eight TBytes (Li et al., 2010), a similar model of the human brain, a “digital twin” of its cellular structure, would be in the range of several PBytes. The interactive exploration (as opposed to simple storage and visualization) of such a dataset is beyond the capacities of current computing, and creates significant challenges in this field (Amunts and Lippert, 2021). Data coming from electron-microscopy, e.g., multibeam electron-microscopy, result, for small samples at nanometer resolution, in comparable data sizes (Eberle and Zeidler, 2018).

Big data problems also appear when moving from single brain data with high spatial or temporal resolution to large cohort studies with thousands of subjects, necessary to address intersubject variability. Large cohort studies are used to study the relationship of structural, functional, behavioral, lifestyle, health and genetic data in thousands of subjects, which are necessary to identify weak factors and their interactions in brain diseases. For example, the UK Biobank provides a unique data set of ∼500,000 participants (Bycroft et al., 2018). Neuroimaging PheWAS was recently introduced as a web-based system to analyze gene-brain relationships, and could be used to study the influences of the apolipoprotein E (APOE) gene on various brain morphologic properties in the Alzheimer’s Disease Neuroimaging Initiative (ADNI) cohort; benchmark tests on the UK Biobank were performed as well (Zhao et al., 2021). The Human Connectome Projects has collected comprehensive neural data and tools, and set a standard in the field (Van Essen et al., 2013).

These and other examples highlight the increasing role of computing, web-based services, and big data analytics in recent brain research. They also illustrate the relevance of large-scale approaches, national and international consortia and research platforms, going beyond research at the level of single labs (Vogelstein et al., 2016). Technically, this is challenging as well: large storage and fast access, as well as powerful computers are required, including high-performance computing. Many applications also need most flexible regimes of work including interactive supercomputing and/or require to execute complex workflows (Amunts and Lippert, 2021). To organize research data in such a way that they are accessible, and well documented, while covering a large spectrum of spatial scales is still a challenge. High-quality solutions have been proposed for dedicated fields of application, e.g., Neurodata Without Borders (https://www.nwb.org/) for neurophysiologicalw and morphologic data at cellular level (Teeters et al., 2015). Another example are tissue models coming from the United States BRAIN Initiative Cell Census Networks (BICCN; https://braininitiative.nih.gov/brain-programs/cell-census-network-biccn), which has started to publish very large datasets of small tissue pieces, but with ultra-high resolution as cell reference atlases Callaway (Callaway et al., 2021). To integrate such information, coming from a multitude of labs, into their spatial, whole-brain context, however, is challenging at the computational and neuroinformatics side.

Box 2: Estimated computational demands to study the human brain

An anatomic 3D model @ 1-μm resolution isotropic needs 2–3 PByte storage per brain

To optimize the computation of fiber tracts with a spatial resolution of 60 μm isotropic would require years for the whole Human brain with current technology

Neuronal network training to extract structural features in images with a spatial resolution of 1 × 1 × 20 μm would require, for the whole brain, 100 d at whole-brain level with current technology

A 10-s point-neuron simulation including 4 million neurons requires 10 min of computation on EBRAINS’ Fenix system (400 core hours)

One second of simulation of a network of 450,000 cells with a high level of details of the hippocampus CA1 region requires at least 20,000 cores and needs 130,000 core hours on the Piz Daint supercomputer at CSCS in Lugano, Switzerland

Simulation of the binding of a single substance at the molecular level with QM/MM (quantum mechanics/molecular mechanics): 20 million core hours on the JUWELS supercomputer at Jülich Supercomputing Centre (JSC), Germany

## The Large-Scale Approach to Advance Neuroscience

Accordingly, several large-scale approaches in brain research have been started to bundle activities (Grillner, 2014). These approaches find a counterpart in other communities, e.g., in the field of astrophysics or climate research, to name only a few of them. Different strategies have been chosen in the brain research community, e.g., addressing the “mind of the mouse” (Abbott et al., 2020), or to map structure and function of neuronal circuits by taking advantage of a non-human primate model, the common marmoset, as in Japan’s Brain/MINDS project (Okano et al., 2015). The United States BRAIN Initiative has an emphasis on the development of technologies to facilitate neuroscience research, and has just recently reported the generation of a cell census and atlas of the mammalian motor cortex; it is argued that a unified and mechanistic framework of neuronal cell type organization integrating multimodal molecular, genetic and spatial information has been established (Callaway et al., 2021). ENIGMA is a global alliance for “Enhancing NeuroImaging Genetics through Meta Analysis” (Thompson et al., 2020). The Human Connectome Projects is providing a large resource of data and tools to explore connectivity of the living human brain (http://www.humanconnectomeproject.org/), that is used worldwide as a basis of studies and experiments. These are only a few examples among several in this field. Comparable approaches can be found in other communities, e.g., biomolecular science (Elixir; https://elixir-europe.org/) and Covid-19 research (Editorial, 2021), but also in other research fields such as particle physics (https://home.cern/). It has been argued that large-scale approaches are influential because they enable investigation of continuously arising new questions from the same data-rich sources and not because they answer any single question (Abbott et al., 2020). At the same time, such approaches were, from their beginning, subject to controversy and criticism (Galison and Hevly, 1992; Mainen and Pouget, 2014).

Another argument for large-scale approaches comes from the high complexity of the research, requiring a collaborative effort over a long time-scale. This is true for research on the human brain. Its complexity, together with major progress in computing, motivated the researchers of the HBP (https://www.humanbrainproject.eu/en/) to initiate a large-scale research project in Europe (Markram et al., 2011). The HBP started in 2013 and was set up to get a deeper understanding of the brain in a time of breathtaking progress in computing and digital technologies (Markram et al., 2011; Amunts et al., 2016, 2019). To achieve this aim, the HBP makes two major innovations. First, a new type of science creating synergy at the interface between empirical research on the brain and advanced computing. And second, an ecosystem and new culture of collaboration leading to substantial progress in our understanding of the brain, brain medicine and brain-inspired technologies.

## EBRAINS Research Infrastructure

**Figure 2.**
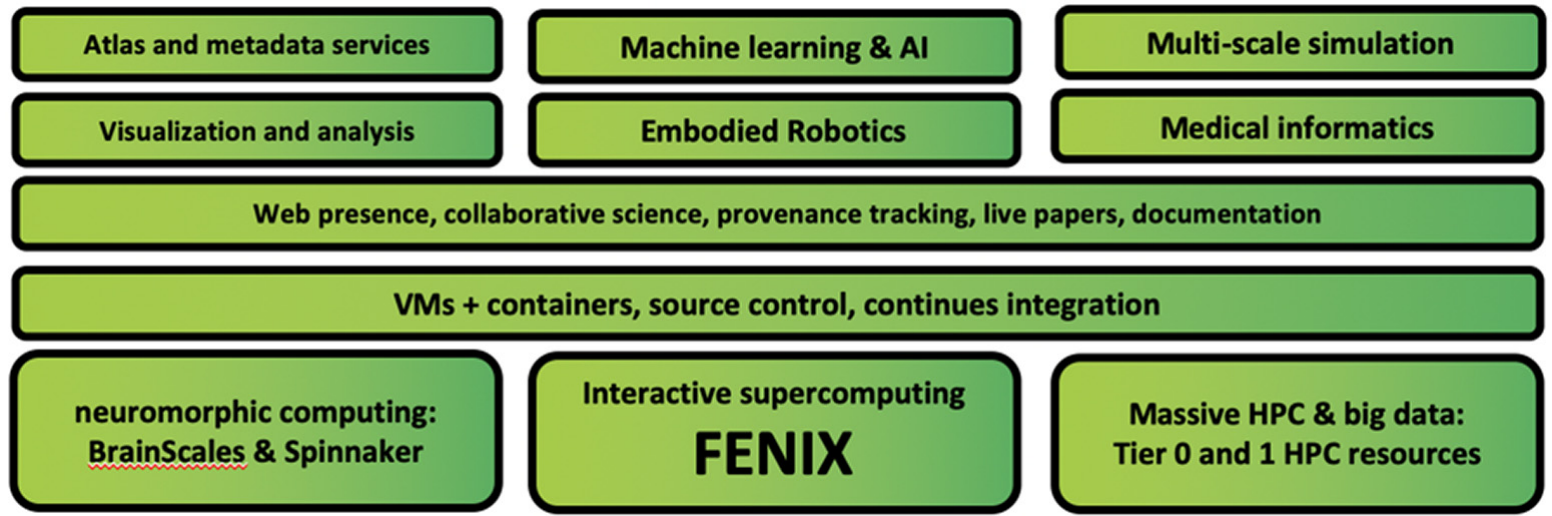
The HBP’s EBRAINS, a research infrastructure providing a broad set of tools and services which can be used to address challenges in brain research and brain-inspired technology (https://ebrains.eu/). The components can be combined resulting in special purpose solutions matching the different research challenges. EBRAINS is offering tools and services in the field of data and knowledge (https://ebrains.eu/services/data-and-knowledge), atlases (https://ebrains.eu/services/atlases), simulation (https://ebrains.eu/services/simulation), brain-inspired technologies (https://ebrains.eu/services/brain-inspired-technologies), medical data analytics (https://ebrains.eu/services/medical-data) as well as a platform for collaboration (https://ebrains.eu/services/community).

Therefore, the HBP decided to develop a distributed, digital infrastructure, EBRAINS (https://ebrains.eu/). It is an open platform for researchers, offering technologically mature tools and services, which is permanently growing and expanding. While being built mainly by partners of the HBP, EBRAINS is increasingly serving the whole science community. It contains different tools and data, which can be combined and linked to each other in a flexible way, allowing researchers to solve their own research questions (Fig. 2). EBRAINS aims to become a powerful resource for the scientific community at large. Many elements of this infrastructure are already in place and can be accessed via its web portal.

EBRAINS is currently used and further developed to advance research mainly in three neuroscience area centered around connectivity: (1) multiscale investigation of brain networks and connectivity; (2) the role of networks in processes underlying cognition and consciousness; and (3) artificial neural networks inspired by the brain, neurorobotics as well as neuromorphic processors, which serve both as accelerators for neuro-derived computation and as tools for neuroscience. A deeper understanding of how neural networks are built and how they function is a basic neuroscientific question of high relevance, and a prerequisite to achieve targeted interventions in brain disease and dysfunction, as well as to develop new diagnostic tools. The perspective of the brain as an embodied network also lets us draw inspiration for technology. New insights into the brain’s information processing and network structure also provide a blueprint for research and development in neuromorphic computing and AI, including deep learning, as well as neurorobotics.

Variations in structure and function between brains are a common thread running through research on connectivity at different spatial scales (Sun et al., 2016; Eickhoff et al., 2018; Larivière et al., 2019; Finn et al., 2020). Intersubject variability can be observed in network organization, including the concentrations of individual receptors, functional connectivity as captured in fMRI, and structural connectivity at different levels. It expresses important properties of the brain linked to resilience against disease, and is an important target of research in itself, providing insights into brain organization (Zilles and Amunts, 2013). The degree to which brains may differ is linked to the genotype, changing during the whole life span and under conditions of brain diseases (Caspers et al., 2014; Thompson et al., 2020). As a consequence, it is necessary for some research questions to study (very) large cohorts and “Big Data” from neuroimaging, genetics, and behavior, to identify single factors and their interaction influencing the brain. The earlier mentioned UK Biobank is an example of a very large cohort, and includes multimodal imaging data, sociodemographic, lifestyle, and health-related information as well as a wide range of physical measures (Littlejohns et al., 2020).

A complementary strategy to consider intersubject differences has been proposed in the context of the Individual Brain Charting Project (IBC), where spatial representations of multiple mental functions are targeted in a systematic and very comprehensive way in a small number of subjects; this also results in large data, because every subject is studied in depth, many times (Pinho et al., 2018). This data set is accessible through the Knowledge Graph and multilevel atlas of EBRAINS (Pinho et al., 2020, 2021b), and can be analyzed in the context of other datasets that EBRAINS is hosting.

Such digital tools and platforms are functioning “stand-alone,” and often have an origin independent from the HBP. However, bringing them together under the roof of the EBRAINS research infrastructure opens up new avenues of application, increases their impact and makes their application more efficient (Fig. 2). This is feasible because EBRAINS is being developed collaboratively by neuroscientists and technology experts in a co-design approach for two reasons: to make sure that it fits the needs of neuroscientists and to ensure that the platform is on a high technological maturity level, user-friendly, and professionally managed. It is also developed collaboratively with philosophers, ethicists, social scientists and public engagement experts, to build a research infrastructure with users that engage with and understand the ethical, philosophical and societal aspects of their work, and an infrastructure that is itself reliably, sustainably, and responsibly constructed and managed.

EBRAINS offers different services (https://ebrains.eu/services/) for curating and sharing data and models, contributing to and accessing brain atlases, using modeling and simulation tools, running closed-loop AI and neurorobotics experiments, retrieving medical brain activity data, and computations based on high-performance computing. The idea behind this is to enable workflows that seamlessly connect elements of the different services. To prove this, so-called showcases have been developed by the HBP (Box 3).

Integrating brain data and knowledge from different research approaches requires curation, proper annotation and provenance tracking. Through the EBRAINS Knowledge Graph, a flexible and scalable metadata management system accompanied by a search user interface, data are made findable, accessible, interoperable and reusable (FAIR; Wilkinson et al., 2016). Knowledge graphs are powerful tools for community-based classification and data aggregation and are also being considered for use in other large brain projects (Yuste et al., 2020). A major challenge for developing a Knowledge Graph is that brain data are massive, complex, semantically and syntactically diverse, coming from many different studies. Accordingly, there is a great need for data and software standards to enable collaboration between scientists internationally (Abrams et al., 2021).

**Figure 3.**
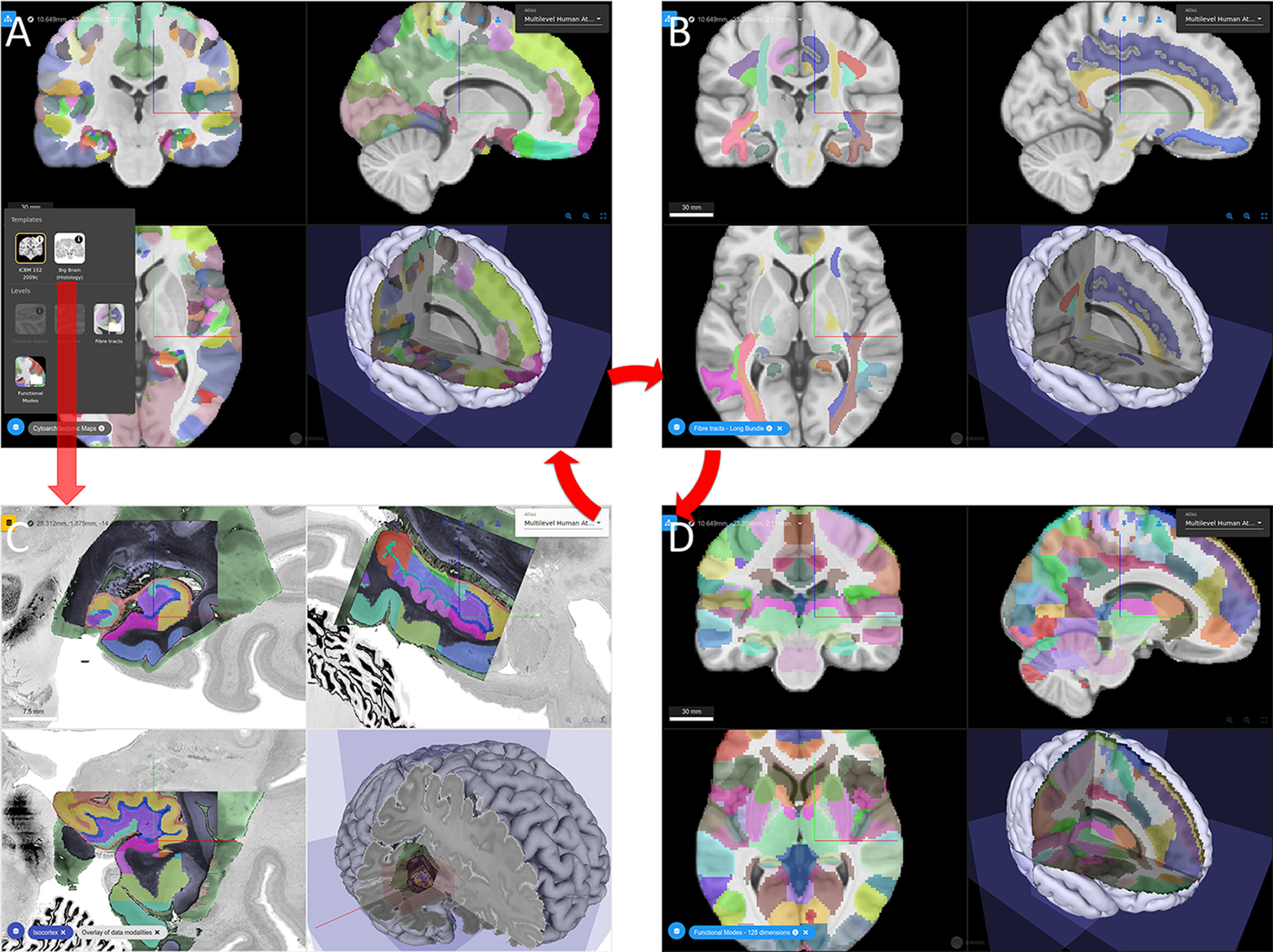
The multilevel Human Brain Atlas provides different maps, e.g., (***A***) Julich-Brain cytoarchitectonic atlas (Amunts et al., 2020), (***B***) DTI-based maps of fiber bundles Guevara (Guevara et al., 2012, 2017), and (***D***) functional parcellation based on task-based fMRI (Pinho et al., 2021a). ***C***, Microscopical data are available through the BigBrain model (Amunts et al., 2013). The atlas provides different types of data in a common spatial framework and allows switching between template spaces.

Brain atlases have a central role to visualize brain data in their spatial context, e.g., to interpret neuroimaging data from living human subject and patients, but also to derive therefrom input for subsequent analysis and model building. Comparative approaches targeting cross-species differences and similarities represent an important field of brain research, but there is still a gap in linking the atlases of the different brains under a common technological umbrella, which creates difficulties, e.g., in understanding homologies. The HBP human brain atlas aims to address this need, and to develop an atlas framework which allows reference to maps of human brain organization, those of rodents, and in the future also monkey brains. The atlas is comparable to “Google Earth,” it allows zooming in and out, the visualization of regions of interest, data extraction from such regions, uploading new maps and results from the user’s own research (Fig. 3). The BigBrain is an anatomic model at 20-μm resolution (Amunts et al., 2013), allowing to map cellular information into a 3D reference space, from cortical layers (Wagstyl et al., 2020) and areas (Schiffer et al., 2021), to volume-of-interests integrated through the VoluBA atlas-tool (https://ebrains.eu/service/voluba/). The latter also opens the perspective to integrate data methods with subcellular resolution, including, e.g., those from electron microscopy, light sheet or two photon imaging. In addition, region-based data, e.g., from multiple receptors of neurotransmitters have been connected to cytoarchitectonically defined areas (Zilles and Amunts, 2009; Palomero-Gallagher and Zilles, 2019). The BigBrain is compatible to atlas data from neuroimaging, and serves as an input for simulation, e.g., using TVB (see Box 3, showcase 1).

Julich-Brain is a part of the Human Brain Atlas and serves as a cytoarchitectonic reference, while taking intersubject variability into account (Amunts et al., 2020). It is linked to a comprehensive map of DTI-based fiber tracts (Guevara et al., 2012, 2017), functional parcellation schemes based on multiple fMRI in a well-defined group of subjects (Pinho et al., 2021a), which provide insights into the cognitive dimension of brain parcellation. MR-based approaches are central to open up applications into *in vivo* imaging, which is relevant for medical research. Being on EBRAINS allows, for example, directly linking information from the atlases with models and simulation. In addition to a web-based viewers, python clients allow a fully programmatic software coupling, e.g., with simulation.

Simulation is increasingly enabled by the computational capabilities and capacities becoming available in Fenix (see below) to handle the very large data representing a human brain, and is in fact driving the development of computer science through its requirements. In the past few years, models of the cerebral cortex (Markram et al., 2015), hippocampus (Coppolino et al., 2021), cerebellum (Casali et al., 2020), basal ganglia (Grillner and Robertson, 2016), typically at the cellular/circuit level, large-scale brain-simulations based on point neurons (Potjans and Diesmann, 2014), or mean-field network modeling (Goldman et al., 2021), as well as models of cognitive functions, such as spatial navigation (Coppolino et al., 2021), object recognition, scene understanding, visuo-motor functions, attention, perception and learning have been developed, and are being constantly improved.

Instead of performing a single simulation, targeted to “fit for everything,” it became evident that various alternative approaches that complement each other, and are becoming more and more interlinked, are the way to proceed (Einevoll et al., 2019). The HBP has made available ∼94 open-source models of neurons and brain circuits. They form reproducible building blocks for more large-scale integrated brain models. Related simulation engines (https://ebrains.eu/services/simulation/) allow the creation of a kind of “digital twins”: from molecular to whole-brain levels. Some models are directly linked to structural information from the brain atlases, and a first multilevel model of a human connectome, capturing connectivity of nerve cells, large-scale fiber tracts and functional neuronal networks, with underlying molecular, cellular and regional brain organization is under development. In parallel, there are also efforts toward cognitive models and (artificial) brain-inspired cognitive architectures are being constructed. Whereas in the past models aimed to reproduce either cognitive processes or physiological brain dynamics, current efforts are directed at models combining both dimensions: cognitive processing in dynamic brain architectures (Jaramillo et al., 2019).

Multilevel simulations for bridging several brain scales are currently realized by coupling simulators for different brain scales, such as single neurons or neuronal populations. Co- simulation technology now enables the synchronous simulation of bi-directionally coupled networks of firing-rate population models (e.g., in the TVB simulator) with regions of individual/networked neurons spiking models (e.g., in the NEST simulator; https://ebrains.eu/service/nest-simulator/). The coupling with other simulators (NEURON and Arbor; https://ebrains.eu/service/arbor/) is a topic of ongoing research.

It has been claimed simulation research represents the next phase of brain research (Fan and Markram, 2019). However, simulation efforts do not replace empirical research, but rather complement it. Ideally, a kind of cross talk can be initiated, with simulation informing empirical research and vice-versa. For example, layer 2/3 pyramidal neurons from the human temporal cortex have a membrane capacitance that was predicted by fitting *in vitro* voltage transients to theoretical transients and then validated by direct measurement in patch experiments (Eyal et al., 2016).

Box 3: Showcases illustrating the applications of EBRAINS for neuroscientific research. All showcases rely on different elements of EBRAINS, and combine different approaches, including simulation, robotics, atlasing, theory, data science, and others (https://www.humanbrainproject.eu/en/science/showcases/)

1. Degeneracy in neuroscience, when is Big Data big enough? Brains are maintaining full functionality within a range of normal variability. Finding out how and which structural changes affect (or not) brain function is an enormous computational challenge. Mastering this challenge will assist in the effort to deliver personalized brain medicine (Jirsa et al., 2017).

2. Improving epilepsy surgery with the Virtual Big Brain. The Virtual Big Brain aims to model and predict activity in an individual patient brain. It links data from high resolution brain mapping to brain avatars, running on high-performance computers to simulate the spread of individual seizure activity along cortical and subcortical surfaces (Proix et al., 2017).

3. Brain complexity and consciousness. Using new methods capable to differentiate states of consciousness from brain activity (Comolatti et al., 2019), and based on EBRAINS, brain simulations of sleep and wake modes have been created Goldman (Goldman et al., 2021). These simulations further the understanding of multiscale brain dynamics of different brain states toward individualized diagnosis and treatment, e.g., in unresponsive wakefulness or locked-in conditions.

4. Object perception and memory. To study perception, a brain-based perceptual-cognitive architecture was integrated in a rodent-like robot. This architecture enables the robot to move around, navigate, remember, and find its way in simple environments. Because of its multisensory predictive coding model (Pearson et al., 2021), it shows enhanced place recognition capacity. These studies pave the way to create brain-inspired robots with perceptually enhanced navigation capabilities.

5. Dexterous in-hand object manipulation. Complex behaviors seem to be built on preexisting, simpler, building blocks (“motor primitives”). To investigate how they emerge, an anthropomorphic robotic hand is trained in several stages using a brain-inspired cognitive architecture. Increasingly complex actions are learned ultimately enabling the model to manipulate objects in the robotic hand. This approach bridges AI, neuroscience and robotics to help to explain why human brains learn skills with much less trials than standard artificial neural networks.

Simulation of human brain models is in most cases extremely compute intensive and requires access to the most recent supercomputing resources. The Fenix infrastructure federates scalable storage and computing resources at multiple leading HPC sites in Europe to provide a single and readily available base infrastructure for data exchange and demanding computational tasks. On top of the Fenix infrastructure, any type of scientific digital service platform can be operated via RESTful APIs (https://fenix-ri.eu/). Fenix that emerged from computer science research in the HBP is an infrastructure-as-a-service (IaaS) for EBRAINS. It has been developed to master the big data challenge of modern brain research. Generic-purpose and domain-specific services provide access to scalable and interactive computing resources via simple-to-use interfaces.

## Digital Tools for Diagnostics and Treatments

Understanding intersubject variability in brain structure, connectivity and signal transduction on the one hand, and the factors modulating it at the different levels of brain organization on the other, is a central question for improving diagnostics and treatment of brain diseases, and key toward personalized brain medicine. Brain diseases represent a major challenge, not only for patients and their relatives, but also in terms of a burden for the health system and more generally, society (Box 4).

Box 4: Brain disorders and their relevance for society

Mental, neurologic, and substance abuse disorders account for more than 10% of global

DALYs

(DALY, or Disease-Adjusted Life Years, is a health metric calculated as the sum of years of life lost and years lived with disability). Six out of the ten disorders with highest DALYs are related to the brain.

Brain diseases represent a considerable social and economic burden in Europe. With yearly costs of ∼800 billion euros and an estimated 179 million (DiLuca and Olesen, 2014) people afflicted in 2010, brain diseases are an unquestionable emergency and a grand challenge for neuroscientists.

Epilepsy is one of the most common neurologic disorders with an estimated prevalence of 50 million worldwide according to the World Health Organization (2020). The complexity of the disease with its vast array of signs, symptoms, and underlying causes of seizures has been challenging to characterize, treat, and understand.

Worldwide, around 50 million people have dementia, with nearly 60% living in low-income and middle-income countries. Every year, there are nearly 10 million new cases. The total number of people with dementia is projected to reach 82 million in 2030 and 152 in 2050 (source WHO, https://www.who.int/news-room/fact-sheets/detail/dementia).

Digital and computational tools are increasingly important in developing new diagnostic tools and options for therapy.

## The Role of Modeling and Simulation in Diagnosis and Therapy

**Figure 4.**
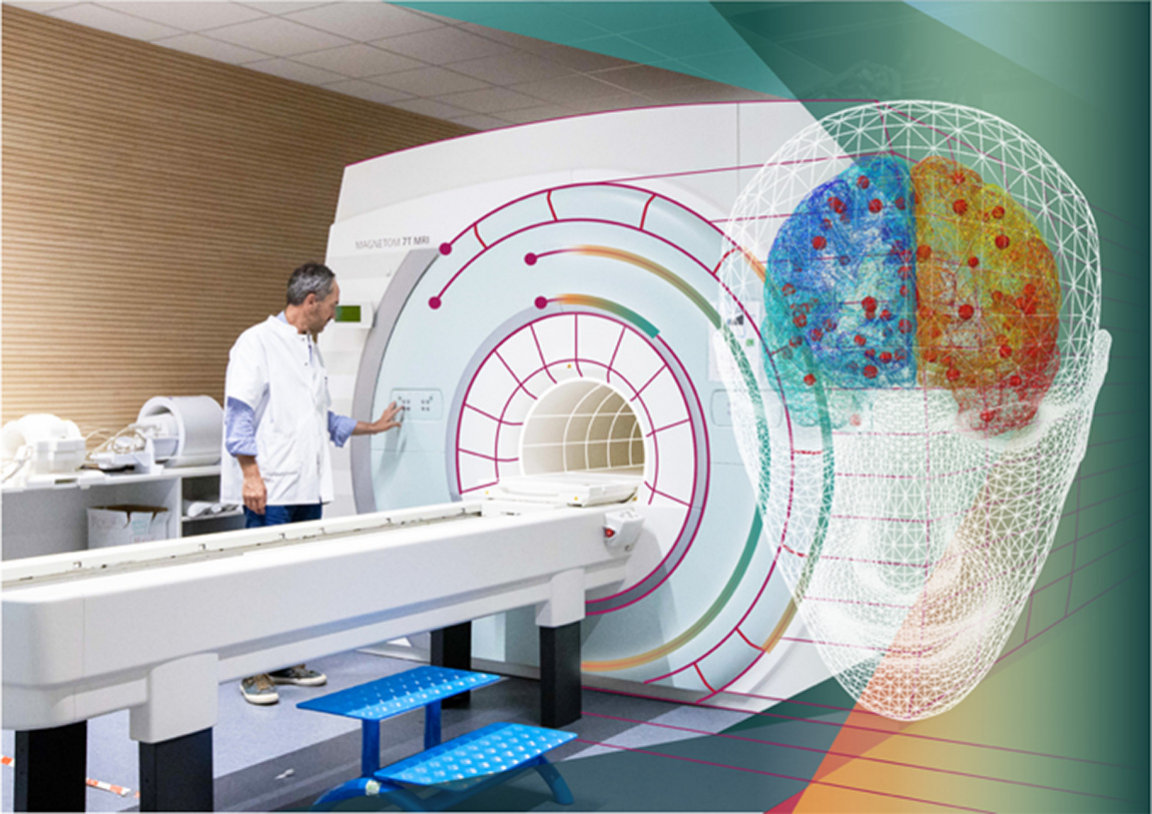
TVB, a data-driven neuroinformatics tool, fusing individual brain imaging data with atlas data and state-of-the-art brain modeling, for personalized simulations of brain activity and clinical interventions. Generative brain models operationalize a causal hypothesis, which is evaluated against the patient’s own brain imaging data using variants of dynamical causal modeling such as Monte Carlo simulations (Hashemi et al., 2020, 2021; Sip et al., 2021; Vattikonda et al., 2021; https://www.humanbrainproject.eu/en/medicine/the-virtual-brain/).

Brain modeling and simulation play an increasing role in the development of new diagnostic and therapeutic solutions. Theoretical concepts built into simulation technologies such as TVB (Fig. 4; https://www.humanbrainproject.eu/en/medicine/the-virtual-brain/) allow the computation of patient-specific brain models serving as *in silico* platforms for clinical hypothesis testing, improved diagnosis, and development of novel interventions (Sanz-Leon et al., 2015; Jirsa et al., 2017). The generative brain models establish a causal hypothesis and are then evaluated against the patient’s own brain imaging data (Friston et al., 2003; Jirsa et al., 2017). For instance, brain regions and fiber tracts serve as stimulation targets in TVB for the study of diagnostic and curative stimulation (Spiegler et al., 2016). “Virtual surgery” can be performed mimicking a patient’s actual surgery and simulating subsequent neural activity on the modified connectome, allowing the optimization of the efficiency of surgical interventions (An et al., 2019; Olmi et al., 2019) and the prediction of surgery outcomes (Aerts et al., 2020). The approach has also been applied to link molecular aspects of neurodegeneration in Alzheimer’s disease with large-scale network modeling (Stefanovski et al., 2021). Modeling and simulation connect the advances in our understanding of brain function to a recent surge in the technological possibilities to write to and read from the brain, bringing together academic researchers, medical doctors and companies to expand the possibilities of linking digital technology to the nervous system and profoundly improve the lives of patients. It has recently been reported that researchers have developed a neuroprothesis for the blind, which was tested in monkeys (Chen et al., 2020). In this experimental study, monkeys were able to recognize different stimuli as simple shapes, motions or letters. The potential applications of brain-machine interfaces are expanding at a rapid pace, prompting the OECD “Science, Technology and Innovation Outlook” (OECD, 2016) to list neurotechnology as one of the ten most promising and disruptive future technologies.

Similarly, the HBP will increase the availability of integrated data and computational models supporting brain state transitions, network complexity and cognitive functions. The PCI is a theory-inspired metric designed to gauge empirically the brain’s capacity for integrating information (Comanducci et al., 2020). The PCI quantifies the algorithmic complexity (information) produced by the causal interactions that are triggered in the brain by a direct cortical perturbation. In practice PCI can be computed by compressing the overall brain electrophysiological response to a direct cortical perturbation with transcranial magnetic stimulation as well as by intracortical stimulation. I.e., the PCI is therefore another example illustrating how knowledge from basic neuroscience is informing theory and modeling, to be transferred into brain medicine.

### The medical informatics platform (MIP)

A MIP (https://ebrains.eu/service/medical-informatics-platform/) enables the analysis of large volumes of patient data throughout Europe (Redolfi et al., 2020). The MIP has opened the possibility to collect data from different hospitals, while considering high standards for data safety and security. It solves the data protection problem: locally installed software allows pooling of preanalyzed data. These data can no longer be assigned to individual patients, but still provide valuable information. For diseases such as Alzheimer’s and Parkinson’s, this enables big-data and AI-driven approaches. Rare diseases with few cases per hospital can thus be analyzed in a statistically valid way. This could bring real breakthroughs, especially for this group, which together account for 20% of all brain diseases.

### The Human Intracerebral EEG Platform (HIP)

Human intracranial EEG data describe brain dynamics with high temporal resolution, and provide unique insights into brain dynamics. At the same time, only a few centers derive such data from patients, and it is still difficult to integrate and analyze such patient data with sufficiently large numbers. The HIP, together with analysis services, is being developed to capture such data (https://www.humanbrainproject.eu/en/medicine/human-intracerebral-eeg-platform/). The idea behind is to pool such data from different sources. This will help to achieve a critical mass of valuable and unique patient data, to enable new clinical analyses based on large cohorts. It will also contribute to basic neuroscience research by providing insights into brain activity and its changes during cognitive tasks.

## Neuro-Inspired Technologies of EBRAINS

Neuro-inspired technologies have a special position among research in the broader field of brain research as they are not only a tool to get new insights into the brain, but are also inspired by brain research to enable new technologies and computing. This includes (1) artificial neuronal networks and AI in general; (2) neuromorphic computing; (3) neurorobotics; as well as (4) high-performance and modular supercomputing. The following paragraphs illustrate some examples.

### Artificial neuronal networks and AI

Considerable progress has been made in implementing artificial neuronal networks, e.g., to classify (medical) images, and to produce *in silico* (cognitive) functions that are comparable to human cognitive functions. Recent progress is made also on applications that are more challenging to teach neural networks such as goal-directed planning, decision-making, and more general problem solving. The way artificial neuronal networks learn, however, currently differs significantly from the way we humans learn. Important aspects of learning in the human brain are not yet well understood, and new mechanisms of learning are discovered, which will further inform such approaches. Only recently, it has been shown that hippocampal output influences memory formation in the neocortex via sensory cortical layer 1 in rodents (Doron et al., 2020). It is expected that a systematic analysis of the differences and commonalities between artificial and natural networks will increasingly contribute to a better understanding of basic neuroscience and information processing, and result in improved concepts derived from large-scale and cellular networks in the brain.

New machine learning algorithms such as e-prop (short for e-propagation) use spikes in their model for communication between neurons in an artificial neural network. The cells only become active when their spikes are needed for information processing in the network. Learning is a particular challenge for such sparsely active networks, since longer observations are required to determine which neuron connections improve network performance. In addition, deep neural networks are by design well-tempered mathematical objects that allow back-propagation of error signals to drive learning through updates of synaptic weights, and spikes introduce discontinuities in neuronal dynamics that preclude the use of similar mathematical approaches (with some possible workarounds (Bellec et al., 2020; Zenke et al., 2021). Whether back-propagation itself is the right approach to capture the essential learning abilities of the human brain has long been an object of debate (Grossberg, 1988). E-prop now provides new solutions by means of a decentralized method, in which each neuron documents when its connections were used in a so-called e-trace (eligibility trace; Bellec et al., 2020). It is speculated that e-prop will drive the development of a new generation of mobile learning computing systems that no longer need to be programmed but learn according to the model of the human brain and thus adapt to constantly changing requirements.

Methods have been proposed to further facilitate learning in recurrent, spiking neural networks, based on a target-based learning scheme in which the learning rule derived from likelihood maximization is used to mimic a specific spatiotemporal spike pattern that encodes the solution to complex temporal tasks (Muratore et al., 2021).

Highly detailed simulations of morphologically realistic, multicompartment neuron models may also yield a unique perspective on the computational limitations of networks built on point neuron models (Gidon et al., 2020), and by extension, of all standard deep neural networks. A new study set out to find a computational method to make highly detailed models of neurons simpler, while retaining a high degree of realism (Wybo et al., 2021). It shows that (back-propagating) action potentials, Ca^2+^ spikes, and NMDA spikes can all be reproduced with few compartments. The study also provides software that automates the simplification, to enable the inclusion of dendritic computations in network models.

In contrast with our everyday experience using brain circuits, it can take a prohibitively long time to train a computational system to produce the correct sequence of outputs in the presence of a series of inputs. By directly following the natural system’s layout and circuitry of the hippocampus, models allow a level of efficiency and accuracy to be reached that opens the way to a new generation of learning architectures, including one shot learning (Coppolino et al., 2021).

The microcircuit of the cerebellum transforms internal signals implementing *de facto* computational algorithms that can be modified through learning. The discovery of adaptable transmission channels supports the long-sought spatiotemporal reconfiguration of the inputs that the cerebellum receives through its numerous sources. This turns into a multidimensional remapping of brain activity that allows the brain to learn from errors implementing sensorimotor and cognitive controllers, and to operate in a predictive manner. The new microcircuit properties are going to be implemented into large-scale models and inserted into closed-loop controllers, neurorobots, neuromorphic computers, and virtual brains, applicable to neuro-engineering, artificial intelligence, and neurology (Casali et al., 2020).

New computational approaches and models are being developed to underpin perception as a learning process in which the brain builds predictions and representations of what causes sensory inputs to arise the way they do (Pennartz et al., 2019a). Basic predictive coding approaches have been extended to large-scale, deep networks trained by Hebbian learning (Dora et al., 2021) have begun to integrate multiple sensory modalities (vision and touch) and have been made more neurobiologically realistic by implementing the principles in single-cell and spiking neural networks (Pearson et al., 2021).

#### Neuromorphic computing

Synergies between advances in brain science and in neuromorphic, brain-inspired computing technologies are currently being explored, showing the potential of these technologies. The high energy consumption of artificial neural networks’ learning activities is one of the biggest hurdles for the broad use of Artificial Intelligence in mobile applications. One approach to solve this problem can be gleaned from knowledge about the efficient transfer of information between neurons in the brain. Neurons send spikes to other neurons but, to save energy, only as often as absolutely necessary.

**Figure 5.**
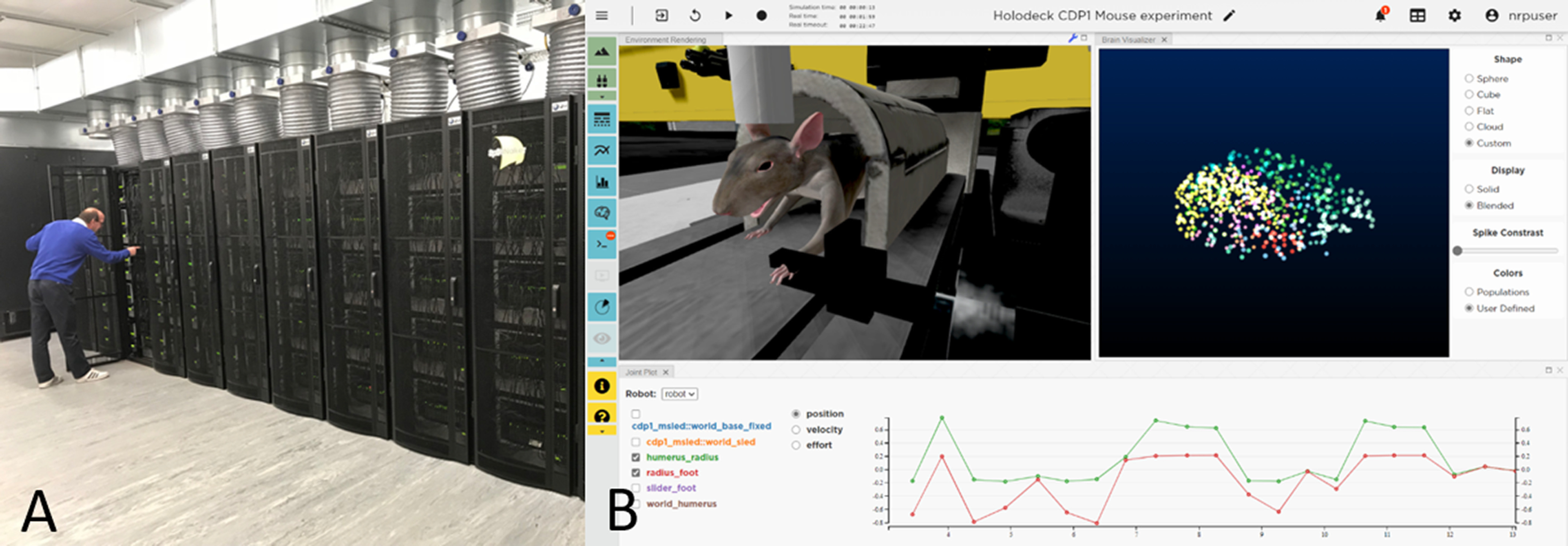
Technologies driven by neuroscience. ***A***, The million-processor SpiNNaker machine at Manchester. ***B***, The user interface of the Neurorobotics Platform NRP, executing the virtualized copy of a real mouse experiment. The mouse body shown in the live rendering on the left is connected to a brain simulation that controls its muscle activations. Body movements are plotted in the graph at the bottom.

Two complementary neuromorphic platforms are offered at EBRAINS as open services (https://ebrains.eu/service/neuromorphic-computing/): SpiNNaker (Furber and Bogdan, 2020) supports very large-scale discrete time numerical simulation. Recent studies have shown that detailed simulations of the cortical microcircuit running on neuromorphic hardware (Fig. 5*A*) can outperform those on conventional machines, in terms of improved throughput and energy efficiency (van Albada et al., 2018; Rhodes et al., 2020).

BrainScaleS supports analog continuous time accelerated emulation, compressing the time-scales required for long-term learning experiments by three to four orders of magnitude. Its modeling capabilities include structured neurons and active-dendrites (Aamir et al., 2018; Billaudelle et al., 2021).

Neuromorphic technology is primed to converge with AI, offering much-needed perspectives in areas where the power demands of even the latest AI-specific chips limit their use at the edge to inference rather than learning. As such, EBRAINS services provides an opportunity for researchers working on this convergence, in the form of a toolchain that connects conceptual exploration to application prototyping and finally implementation. Edge computing applications are poised to benefit most from the emergence of neuromorphic chips capable of both energy-efficient, low-latency processing of data streams and concurrent learning based thereon. Autonomous robotics will also greatly benefit from such chips, insofar as they are in all likelihood key enabling technologies toward the implementation of complex cognitive functions such as decision-making, situational awareness, contextual adaptability, etc. Understanding how those arise from the human brain, both at the computational and implementation level, is a challenge taken on by the HBP.

#### Neurorobotics

Modeling how the brain is situated in a specific environment with which it interacts through its body is mandatory for understanding how neural activity and physical behavior give rise to each other. In line with the position of enactivism, embodied modeling of perception and cognition stresses that actions of the body endow the brain with causal power in the world and that any neuronal network likely serves the purpose (directly or indirectly) to enhance successful interaction with a complex, dynamic, environment. Neurorobotics provides both the tools and the theory for embedding brain simulations into robotic bodies to establish a closed loop of perception, cognition and action between the brain, its body and the environment (Fig. 5*B*). This makes it possible to not only create highly detailed models of the brain’s structure but to also reproduce the dynamics that emerge from them under highly realistic conditions.

The Neurorobotics Platform (https://neurorobotics.net/) of the HBP (Falotico et al., 2017) provides an integrated cloud-based simulation framework for the design and execution of virtual neurorobotics experiments in physically realistic environment models (Fig. 3*B*). The platform is able to run large-scale spiking neuronal networks implemented with the NEST simulator on supercomputers on the order of millions of neurons, billions of synapses (Helias et al., 2012), and supports modular, heterogeneous control architectures for the simulated agents. It is also accessible via https://ebrains.eu/service/neurorobotics-platform/.

As the Neurorobotics Platform contains simulation models and tools required to replace all components of traditional neuroscience experiments by digital twins, it lays the foundations for virtualized neuroscience. Fully virtual experiments cannot only reproduce previously achieved findings from the lab but importantly also predict new results at high speed and low cost. The more these predictions are refined by subsequent experimental ground truth, the better future predictions get. This makes research not only more efficient but considerably enlarges the exploration space.

Another major advantage of virtual neuroscience is that the full state of the experiment from the activations of muscles to the firing of individual neurons is observable any time at any desired level of detail. This enables a new form of real-time brain atlases where not only the brain’s structure can be observed but also its live activity. These atlases therefore not only represent space but also time.

Closed-loop neurorobotic systems are not constrained to virtual experiments. They can also be set up in the real world by connecting a brain simulation to a physical robot. In particular, neurorobotics allows for embodiment of cognitive architectures on anthropomorphic robots thus enabling the transfer of emulated human capacities to artificial agents. The adaptive “brains” of these robotic agents are amenable to close scrutiny, and inspecting how they solve goal-directed tasks may inspire new testable hypotheses whether the human brain has developed similar representations and processes (Kroner et al., 2020). Neuromorphic computing is an essential prerequisite for these studies because the simulation of the neural models needs to run in real time. This makes neurorobotics an ideal tool to prototype applications that embed neuromorphic computing at their core, but also rely on complementary, more standard technologies. Such prototyping is made all the easier by the fact that the Neurorobotics Platform can natively use neuromorphic hardware as a simulation backend and will also be enabled in the future to perform hardware-in-the-loop simulations.

Building adaptive biologically inspired cognitive architectures contributes to our understanding how the brain works by emulating some aspects of its functions. For example, large-scale neural network models are created that are themselves composed of smaller neural network modules that correspond roughly to specific brain areas. These types of architectures enable the development of new types of training protocols and the investigation of long-standing questions such as the separation problem and the binding problem (von der Malsburg, 1999). Neurorobotics therefore not only provides the foundations for virtual neuroscience but also enables effective knowledge transfer to artificial intelligence and machine learning.

## High-Performance and Modular Supercomputing

While neuroscience in the past rather rarely required extreme-scale computing, the need to simulate at large scale or to process and analyze datasets in the PByte range has changed the situation (Amunts et al., 2014; Einevoll et al., 2019; Menzel et al., 2019; Rossetti et al., 2019; Franceschini et al., 2020; Amunts and Lippert, 2021) and motivated the development of the federated Europe-wide HPC infrastructure Fenix (https://fenix-ri.eu/). Meanwhile, a strong community has emerged to drive such development, and Fenix resources are openly available for compute and storage intensive projects. The methods that are being developed in this context often go beyond neuroscience, and are open to other research communities. Both edge computing and cloud computing are considered for use cases from neuroscience. The HBP is developing tools for interactive supercomputing, web-based visualization and analysis of big data in the context of Fenix. Researchers are preparing use cases for Exascale performance on modular supercomputers to be built in 2023/24 under the umbrella of the EuroHPC Joint Undertaking and participating countries to coordinate their efforts and pool their resources in Europe to enable world-class Exascale supercomputers, together with researchers from other communities. Joint interests in the development of high-performance computing, its hardware and software, will open new perspectives for collaborative project across different research domains.

## Collaborative Perspectives

In the middle and long run, the aim is to further develop EBRAINS as a global platform for collaboration and exchange among researchers, a mechanism for users to participate in the development of new tools, methods, and to provide and exchange their data. Such digital research infrastructure is not only relevant for individual collaboration between researchers, but also between large-scale initiatives, e.g., the United States BRAIN Initiative, with initiatives such as Healthy Brains for Healthy Lives (HBHL) in Canada, and brain initiatives in China, Japan, Australia, to name some of them. For example, the Canadian-German collaboration HIBALL (https://bigbrainproject.org/hiball.html) focuses on the BigBrain as a high-resolution model of the human brain (Amunts et al., 2013) to reinforce utilization and co-development of the latest AI and high-performance computing technologies for building highly detailed 3D brain models, and connects EBRAINS and HBHL. It provides next-generation brain models, integrates multimodal data to the BigBrain, takes care about interoperability of scientific workflows, and develops new deep neural network architectures. It has built an active community in a short time that uses and further develops tools for brain research. Such synergy became feasible also because it can build on existing infrastructures both in Canada and Europe. It would also be a tool that can be used to link ultra-high-resolution models of volume of interest such as developed in the BRAIN Initiative Cell Census Network, e.g., from the primary motor cortex (Callaway et al., 2021). This would have the advantage of integrating highly detailed, multimodal information into its spatial context, thereby linking advantages of the bottom-up with the top-down approach.

Several brain initiatives have founded the International Brain Initiative (IBI; https://www.internationalbraininitiative.org/) to join forces. As an integral part of the science and technology agenda, IBI addresses questions of ethics, philosophy and society. Specifically, at the interface of neuroscience and technology, the clinic and society, new challenging issues arise, including, for example, data protection and privacy, pharmacological and digital neuroenhancement, and dual use of brain-related technologies (Salles et al., 2019a; Flick et al., 2020). Another new field is concerning the ethics of AI, which plays an increasing role (Stahl, 2021). All these questions have in common that they cannot be answered by a single discipline, but require a cross-disciplinary interaction and broader discussion in society. Technical advances need to be delivered in a way that reflects European values and principles, such as non-discrimination, fairness and privacy. Ethical considerations like these are an integral part of technology developments in EBRAINS. Through the efforts of the HBP, EBRAINS is intended to integrate neuroethics and philosophical analysis to enhance the neuroscientific work (Evers, 2009; Salles et al., 2019a, b). Philosophical analysis provides clarification of scientific concepts such as behavior, intelligence, digital twin and consciousness and explores how neuroscientific knowledge is constructed, what are its underlying assumptions and how they are justified, how results may be interpreted, and why or how empirical knowledge of the brain can be relevant to philosophical, social, and ethical concerns (Pennartz, 2015; Salles et al., 2019b).

Conceptual clarification and analysis are the basis for addressing more practical issues raised by neuroscientific research from data protection autonomy and identity concerns (Amadio et al., 2018). EBRAINS is expected to adopt an inclusive and co-creative way of working, engaging with multiple audiences and communities to discuss ethical issues, developing novel insights into responsible innovations and their clinical and societal applications (https://ebrains.eu/discover/).

## Conclusions

Gaining a comprehensive understanding of the human brain, its connectome, and parcellations requires understanding the multilevel organization of the brain as an embodied network and complex system enabling perception, action, consciousness and cognition. Combining the perspectives of multilevel brain organization with embodiment is not only relevant to capture the full scope of brain diseases and to be able to develop new therapies, but also for the development of neuro-inspired technologies, and future neurorobotics.

There is an urgent need to accelerate efforts for mental and brain health by making full use of insights from brain research and modern digital tools. Based on use cases from neurology already available in EBRAINS, including the MIP and the Human Intracerebral EEG Data Platform, the HBP-built research infrastructure is now being further developed to support research in mental health, psychiatric disorders, neurosurgery, and neuroradiology, but also more broadly in the medical field.

Insights into fundamental questions of brain organization will provide the key to new computing technologies, artificial neuronal networks, cognitive computing and neurorobotics as an integrative overarching technology both for experimentation and for substantially advancing real robotics. Making such technologies more “neuro-inspired” is expected to significantly speed up their development. Neurorobotics and neuromorphic computing will benefit from being increasingly neuro-inspired.

The amount of brain data is increasing rapidly. The effort in terms of time, knowledge and methodology needed to make it FAIR has long been underestimated and resources should be planned, from the very beginning of each research project, to address this.

The Human Brain Atlas allows access to multiple brain data according to their spatial organization through viewers, but also fully programmed software coupling. This is a potential game changer for analyses of big and complex data on systems of the highest performance, but also for modeling and simulation, which become biologically more realistic.

Modeling and simulation have started to develop from different angles, and they used different approaches. But now we are in a position where we can link them, which enables bridging the different scales, to better constrain and to verify results of simulation.

Collaboration across boundaries of institutions, sectors, nations, research disciplines and cultures is indispensable for progress in neuroscience. Moreover, insights from brain research will increasingly influence learning and education and have an impact on our society.

To stay ahead of emerging ethical, societal and legal issues, and to strengthen the societal benefit and acceptability of its findings, EBRAINS need structures and strategies for engaging in dialogue with communities on issues of immediate and long-term relevance, including data ethics, neuroethics, animal use and well-being, dual use, gender equality, and diversity.

The culture of collaboration in the neurosciences is changing. The authors are convinced that we can contribute to making it more open, cooperative, and participatory, for the benefit of neuroscience, medicine, and society, which marks the beginning of a new paradigm for understanding the brain.

## Synthesis

Reviewing Editor: Christoph Michel, Universite de Geneve Decisions are customarily a result of the Reviewing Editor and the peer reviewers coming together and discussing their recommendations until a consensus is reached. When revisions are invited, a fact-based synthesis statement explaining their decision and outlining what is needed to prepare a revision will be listed below. The following reviewer(s) agreed to reveal their identity: Karl Friston, William Stacey.

We apologize your manuscript suffered some delay. One reviewer never sent the report and after multiple deadline extensions, I decided to move decision forward by inviting a third reviewer. Your manuscript was thus evaluated by R1 and R3.

We all feel there is value in your proposal, but we also agree that the current version requires improvement. While we appreciate the vision of EBRAINS to theoretical and methodological integrate knowledge for a more intuitive understanding of the brain, we feel the approach still remains somehow unguided. We also feel that including perspectives from different co-authors may have resulted in a less coherent narrative. I copy below the comments that emerged along the review and consultation session, which you need to address carefully in a revised version.

1- We feel your report misses discussing a conceptual scaffold for thinking about functional architectures in the brain at different scales, which may help to engage potential users of EBRAINS. Here are some examples, but please feel free to adapt these suggestions or to use other examples as you wish.

In systems neuroscience, particularly in imaging neuroscience, it is usual to start with some basic principles of functional brain architectures. We would recommend something along the following lines:

"In systems neuroscience, particularly imaging neuroscience, people generally appeal to two principles of brain organisation: namely, functional specialisation and functional integration. This distinction inherits from the early localizationist work of 19th-century neuroanatomy and neuropsychology - complemented by studies of disconnection and, in the 21st-century various kinds of connectomics. In brief, functional specialisation refers to...”

It will be good here to talk about 'structure-function relationships' and how cytoarchitectonics and receptor mapping underwrite functional specialisation or, indeed, segregation at various anatomical scales.

"Functional integration refers to the coordination of distributed neuronal responses; often cast in terms of connectivity and connect terms....”

It would be useful here to talk about connectivity at a molecular, electrochemical and neuronal level, trying to abstract the underlying principles. One key thing - that has dominated research in systems neuroscience - is the distinction between functional connectivity and effective connectivity; namely, detecting statistical associations as a prelude to drilling down on the causal or directed connectivity - usually in the form of state space models based on differential equations.

Finally, we feel the introduction needs to set up the notion of a hierarchical architecture; both in terms of anatomical connectivity and effective connectivity (e.g., the distinction between drivers and modulators). This is necessary because notions of top-down and bottom-up (referred to in the body of the text) are only defined in relation to a hierarchical organisation.

2- At another level, one of us feels the paper should provide a perspective of how EBRAINS has evolved from the early days of the Human Brain Project to move the field forward both as a resource and a vision. In this spirit, it may be helpful closing with a series of questions that potential collaborators and contributors may be asking themselves, and so they may generate debate. These questions are not exhaustive but speak to the sort of issues that could be debated to inform the future trajectory of EBRAINS. The reviewer provides some suggestions:

a) Is EBRAINS too heavily reliant on emergentism? In other words, many of the examples that we have used to showcase various aspects of EBRAINS rest upon the assumption that self-organised behaviour will emerge from assembling suitably realistic and complex elements of neurons, neuronal populations and circuits. Is this a tenable position? The converse position rests on a first principles account of functional or computational architectures that holds at multiple scales. Perhaps the best example of this is the free energy principle that uses the same principle to explain self-organisation at multiple scales, ranging from a single dendrite, through to multicellular organisation and message passing on large graphs1-3. From the perspective of EBRAINS, the choice here is whether to use the data and machinery on offer to build ever larger circuits and graphs in the hope that neurobiologically plausible behaviours will emerge. The alternative approach would be to define behaviour in terms of a normative or optimisation perspective and then seek evidence for the computational realisation of optimisation processes - and their neuroanatomical and neurochemical structures - in the data.

b) Is big data science necessary? The way EBRAINS is portrayed is to accommodate the wealth of data necessary for building bigger brains and coupling different scales or levels of dynamics. But is this necessary? The counter position would argue that advances in physics have not used supercomputers. They rest upon mathematical or conceptual devices to abstract the behaviours that matter. These range from phase-reduction techniques and mean-field approximations through to the apparatus of the renormalisation group. Although sometimes abstract in nature, these are powerful approaches that offer more plausible descriptions of self-organisation across scales. Interestingly, for things like the renormalisation group to work, one has to cast brain function as an optimisation process with an associated Lagrangian or dynamics that is conserved over scales. The counter position to big data science then becomes extracting key quantities at one level of analysis that inform - and are informed by - variables at the next. From the perspective of EBRAINS, this raises the question: should EBRAINS try to accommodate all data or focus on the best kind of data at each level of description.

c) Has EBRAINS forgotten about enactivism? This question is particularly precedent given the recent turn in systems neuroscience and ethology towards enactivism, embodiment and situated cognition. Many of the neuroscience initiatives currently supported by EBRAINS may not be sufficiently sensitive to the embodied approach required by a more ethological approach or, indeed, neurorobotics. A good example of this is predictive processing. Predictive processing now predominates in the cognitive and systems neurosciences with a broad uptake, explanatory scope and power. Yet, from the perspective of EBRAINS it features only as a representationalist implementation of predictive coding. It has been argued that representational approaches reflect a 20th-century style of thinking, in which information is extracted from sensory input to form representations. This contrasts with 21st-century views of a more constructivist kind. Here, top-down influences are not just there to contextualise hierarchically lower-level processing, it is there to supply predictions that are constructed by high level representations. Crucially, these predictions also prescribe movement in embodied brains. Does this need to be an explicit part of EBRAINS? Does EBRAINS need to include kinematics and motor control that handshakes with robotics?

d) Where are the generative models? This question relates largely to the computational neuroscience in EBRAINS. Broadly speaking, one can use computational models to simulate biologically plausible behaviour. Alternatively, one can use computational models as observation models that are fit to biological data. These observational models effectively generate the data, one would observe if the implicit generative model was correct. Having an explicit generative model at hand is potentially important because it enables one to treat the generative model as a hypothesis - and evaluate the evidence for different models given some data. This can have profound implications for data analysis. A nice example of the distinction between classification (a.k.a. recognition) and generative models, as afforded by the perturbation complexity indices used to assess effective connectivity and variants of dynamic causal modelling used in the virtual brain project. The first is an example of trying to recognise patterns or signatures in the data that have some predictive or construct validity in relation to nosology or different brain states (e.g., sleep or levels of consciousness). The second approach uses the data to optimise the structure of - and parameters of - some hypothetical network model. From the perspective of EBRAINS are, the implications of this distinction speak to the focus on the inversion or optimisation of generative models versus the classification ethos inherent in data mining. A nice example of a generative model would be the notion of a 'digital twin', whose parameters are optimised to best explain personalised data in EBRAINS - as a prelude to characterising within and between subject variability.

Suggested references:

1 Kiebel, S. J. & Friston, K. J. Free energy and dendritic self-organization. Front Syst Neurosci. 5, 80 (2011).

2 Friston, K., Levin, M., Sengupta, B. & Pezzulo, G. Knowing one's place: a free-energy approach to pattern regulation. J R Soc Interface 12, doi:10.1098/rsif.2014.1383 (2015).

3 Friston, K., Parr, T. & de Vries, B. The graphical brain: Belief propagation and active inference. Network neuroscience (Cambridge, Mass.) 1, 381-414, doi:10.1162/NETN_a_00018 (2017).

3-We feel to some extent the paper primarily reads like a list of accomplishments of the HBP. While this is not necessarily bad, we feel there is value in moving debate to what is coming with EBRAINS instead. For instance, the need for a “culture change” is an important issue the paper should further develop to mitigate the focus on HBP. This may be a section of the discussion. What is needed to move this forward? What is the change? How will it be accomplished? Some specifics should be mentioned, e.g. pooling of data to make large EEG papers rather than individual ones.

4-We are not sure what is the difference between HBP and EBRAINS, which means the text needs some clarification. Also, the scope is so vast that most subjects are barely mentioned, let alone explained. Then it is hard to know when the citation/link is meant as a mere citation versus as a pointer to use the tool. We recommend a figure/table that summarizes all the different options that this paper “links” to, organized similar to a flow chart. Perhaps using the same headings as the paper itself. That would be a cogent resource to find the tools.

5-We recommend the title to mention EBRAINS, since that is really what this paper is about. We also feel that the abstract reads a bit messy. The mention to BigBrain for instance, is distracting.

6- On page three, the discussion about “top down” approaches mentions only a single citation, and does little to explain what it is. That paragraph could be supplemented with some other citations and explanation, i.e. what does “top down” mean? What are some ways it is implemented?

7-Please, provide a visual abstract
